# Levels and determinants of maternal mortality in northern and southern Nigeria

**DOI:** 10.1186/s12884-019-2471-8

**Published:** 2019-11-12

**Authors:** Catherine Meh, Amardeep Thind, Bridget Ryan, Amanda Terry

**Affiliations:** 1 Department of Epidemiology and Biostatistics, Western University, 1151 Richmond St., London, ON N6A 5C1 Canada; 2 Department of Family Medicine, Western University, 1151 Richmond St., London, ON N6A 5C1 Canada; 3 Schulich Interfaculty Program in Public Health, Schulich School of Medicine and Dentistry, Western University, 1151 Richmond St., London, ON N6A 5C1 Canada

**Keywords:** Nigeria, Maternal mortality, Maternal health

## Abstract

**Background:**

Maternal mortality is still a major risk for women of childbearing age in Nigeria. In 2008, Nigeria bore 14% of the global burden of maternal mortality. The national maternal mortality ratio has remained elevated despite efforts to reduce maternal deaths. Though health disparities exist between the North and South of Nigeria, there is a dearth of evidence on the estimates and determinants of maternal mortality for these regions.

**Methods:**

This study aimed to assess differences in the levels and determinants of maternal mortality in women of childbearing age (15–49 years) in the North and South of Nigeria. The Nigeria Demographic and Health Surveys (2008 and 2013) were used. The association between maternal mortality (outcome) and relevant sociocultural, economic and health factors was tested using multivariable logistic regression in a sample of 51,492 living or deceased women who had given birth.

**Results:**

There were variations in the levels of maternal mortality between the two regions. Maternal mortality was more pronounced in the North and increased in 2013 compared to 2008. For the South, the levels slightly decreased. Media exposure and education were associated with maternal mortality in the North while contraceptive method, residence type and wealth index were associated with maternal death in the South. In both regions, age and community wealth were significantly associated with maternal mortality.

**Conclusions:**

Differences in the levels and determinants of maternal mortality between the North and South of Nigeria stress the need for efforts to cut maternal deaths through new strategies that are relevant for each region. These should improve education of girls in the North and access to health information and services in the South. Overall, new policies to improve women’s socioeconomic status should be adopted.

## Background

Maternal mortality continues to claim the lives of women of childbearing age worldwide. This problem remains a challenge for many countries that still struggle to prevent it. Over half a million annual maternal deaths propelled maternal mortality onto the international stage, where it became a global priority and the chosen outcome to assess progress on maternal health [1]. Still, an estimated 810 maternal deaths occur each day in the world [2]. Maternal mortality adversely affects women, their families and communities. The Millennium Development Goal (MDG) 5 to reduce the global burden of maternal death by 75% by 2015, and the recent Sustainable Development Goal (SDG) 3, which seeks to significantly cut the number of deaths to 70 per 100,000 livebirths by 2030, led to the implementation of interventions to reduce the global burden of maternal mortality.

Many countries over the last three decades have reduced their maternal mortality levels and contributed to the global decline of maternal deaths. However, in sub-Saharan Africa, where over 50% of all maternal deaths occur, maternal mortality rates have largely stagnated. Hemorrhage, abortions, sepsis and obstructed labor are some of the leading causes of maternal death in this region [3]. While countries like Rwanda - which once had the highest level of maternal deaths in the world (in 1995, maternal mortality ratio (MMR) was 2300/100,00 livebirths) [4, 5] – have met their MDG 5 target of reducing maternal deaths [6] to 320/100,000 livebirths [7], other countries in Sub-Saharan Africa either had no significant change or experienced an increase in their levels of maternal mortality, even with implementation of evidence-based interventions. By the close of the MDG period, countries such as Chad (980/100,1000), Central African Republic (880/100,000), and Burundi (740/100,000) had recorded the highest MMRs [7].

### Maternal mortality in Nigeria

Nigeria is one of the countries in Sub-Saharan Africa where maternal mortality has remained a problem. The country’s progress towards cutting the number of maternal deaths has been largely insufficient [6]. Maternal mortality persists in Nigeria despite strategies like the promotion of institutional deliveries, training and deploying new skilled health workers. It is also among the top six countries in the world that contribute to more than 50% of all global maternal deaths [8]. In 2008, Nigeria had the second largest recorded number (50,000) of maternal deaths with an estimated MMR of 840/100,000 livebirths [9].

The Nigeria Demographic and Health Surveys (NDHS) revealed a national MMR of 576 deaths per 100,000 livebirths and 545 deaths per 100,000 in 2013 and 2008 respectively [10]. However, studies have shown that the levels of maternal mortality vary within the country. There are states and health facilities that have higher levels of maternal mortality compared to the national average. For instance, some northern states like Kano in 2008 had an MMR of 1600 deaths per 100,000 livebirths [11] while 1049 deaths per 100,000 livebirths were reported in Zamfara state [12]. Also, health facilities show similarly high levels of maternal mortality with 927 deaths per 100,000 livebirths reported for 21 health facilities in three states - Katsina (North), Lagos (South) and the Federal Capital territory (North) [13].

Like many countries in Sub-Saharan Africa, the leading causes of maternal death in Nigeria are obstetric hemorrhage, eclampsia, sepsis and complications from unsafe abortions [14, 15]. Similarly, studies show that factors such as age [14], education, antenatal care, parity [13], domestic violence and social autonomy [16] (which have been established as determinants of maternal mortality) are associated with this outcome in Nigeria.

### Nigeria: North and South

Nigeria is the most populous country in Africa and came to be a nation through the unification of two separate British northern and southern protectorates consisting of six geo-political zones: in the North, North West, North Central, North East; and in the South, South East, South-South and South West.

The National Population commission of Nigeria shows the North and South as two distinct regions [17]. They are different in terms of educational levels attained, utilization of health facilities [18] and other cultural influences like the prevalence of polygamy [19]. These factors are linked with health outcomes such as maternal mortality. The Multiple Cluster Indicator Survey of 1999 revealed some variation in the levels of maternal mortality between zones in the North and South. In the South-West, MMR was 166 maternal death per 100,000 livebirths compared to 1549 maternal deaths per 100,000 livebirths in the North-East [11, 15].

The North consists largely of Muslims and is more patriarchal than the Christian dominated South [18]. Women in the North are less likely to give birth at health facilities [20] and many in some northern states, live far from health centers which are plagued by severe shortages of health workers [18] compared to the South of Nigeria.

Other North-South differentials exist in Nigeria. In terms of the probability of childhood mortality, women in the North experience higher levels and have a higher likelihood of having experienced previous childhood mortality than their southern counterparts [17]. The use of contraceptives was higher in the South of the country compared to the North [21]. This gap in contraceptive use between the North and South is attributed to socioeconomic and ideational (ideal number of children, awareness of contraceptive method, and self-efficacy) factors [22]. Meanwhile, northern residents were more likely to live within 5 km radius of health facilities or outreach posts that offer immunization services than those in the South [23].

### Current study

It is against this background that the North is said to have had a worse performance than the South on maternal mortality [24] and other health outcomes. The disparities in these regions influence their levels and determinants of maternal mortality. Yet, there is a dearth of literature on the estimates and determinants of maternal mortality for the whole of North and South Nigeria. To date, maternal mortality data for the North and South have been extrapolated from individual states’ community and facility data. Hence, the aim of this study was to assess the levels and determinants of maternal mortality in the Northern and Southern regions of Nigeria among women of childbearing age using Nigeria Demographic Health Surveys.

## Method

### Framework

A modified conceptual framework based on the work of McCarthy and Maine was used to analyze the determinants of maternal mortality in the North and South of Nigeria. [25]. This incorporates the roles of socioeconomic, cultural, behavioral and biological factors on maternal mortality, thereby making it the most inclusive framework for assessing determinants of maternal mortality. It is also a widely used framework in studies about factors that affect maternal health [26–28].

**Fig. 1:**
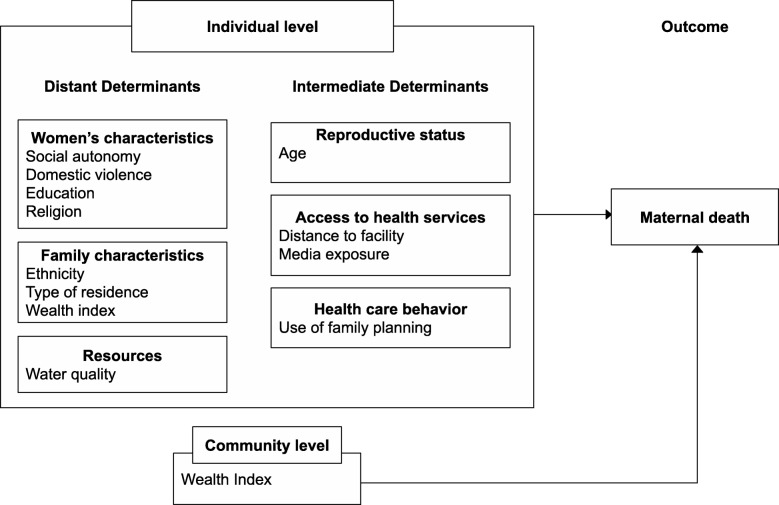
Adapted framework (McCarthy & Maine) determinants of maternal mortality

Each determinant of maternal mortality in the modified framework is classed either as intermediate or distant based on its proximity to the outcome. Intermediate determinants (Fig. 1) have a more direct influence on maternal mortality while distant determinants impact the outcome by way of their influence on the intermediate factors. The modified framework distinguishes between determinants that impact maternal mortality on the individual or community level.

### Data source

The NDHS are conducted by the National Population Commission of Nigeria in partnership with USAID and technical support from local government to gather reliable and representative data on population, health, and nutrition indicators on average, every 5 years in Nigeria. The population of interest in these surveys are men and women between the ages of 15 to 49 years. The NDHS of 2008 and 2013 are the most recent national surveys and were selected for this study. These two cycles allow for the regional levels of maternal mortality to be assessed over time. Given the rarity of the outcome for this study, it was necessary to increase the study power to detect associations between maternal mortality and the independent variables at the regional level. To achieve this, the two survey cycles were pooled to increase the sample size notably, the number of maternal related deaths.

### Study design

This study assessed the determinants of maternal mortality by using a cross-sectional study design and analyzing secondary data for women of childbearing age (15–49 years) that were part of the NDHS as respondents or were identified as deceased siblings (cases of maternal mortality) using the direct sisterhood method (a variant of the sisterhood method [29]). This method identifies deceased siblings through select questions asked of the respondents and is widely used by the Demographic and Health Survey Program [30] and maternal health studies [31, 32] to obtain periodic estimates of maternal mortality.

### Study population

This study focused on women who had been pregnant and had given birth to at least one child in Nigeria. Hence, nulliparous women (women with zero births) were excluded. The study participants could either be respondents (living women) or deceased women (respondents’ siblings) who were cases of maternal mortality that were identified through the direct sisterhood method.

#### Respondents (living women)

A respondent in the NDHS was included in this study if she had a total of one or more children ever born.

#### Deceased women (respondents’ siblings)

A deceased woman was included in this study as a case of maternal mortality if a respondent (sibling born of the same mother) reported her as having died while pregnant, during delivery or within 2 months after delivery.

Age at death (NDHS 2008 and 2013) and parity (NDHS 2013) were recorded for the deceased women. Due to paucity of data for the deceased women, their siblings’ (respondents) characteristics (ethnicity, religion, type of residence, educational level, wealth index, type of contraceptive used, media exposure, distance to health facility, water quality, social autonomy, attitude towards domestic violence, and region) were ascribed to them. We based this on the assumption that deceased siblings and respondents share similar characteristics and evidence which supports this approach [33, 34].

### Variables

#### Dependent variable

The outcome variable for this study was maternal death. This status was assigned to a deceased woman if a respondent indicated “yes” to whether her female sibling died while pregnant, during child birth, or within 2 months of delivery. The dependent variable was coded as binary; cases of maternal mortality (deceased siblings) were coded as 1 and 0 was assigned to living women (respondents).

#### Independent variables

##### Individual level

***Intermediate***

Age was recoded in years and treated as a continuous variable. Difficulty accessing healthcare services was assessed with the variable “distance to health facility”. The response options included two categories - “small problem”, and “big problem” with distance to health facility. A composite variable for media exposure was created using three measures: frequency of listening to the radio, watching television, and reading a newspaper or magazine. These were scored (0–9) and categorized as no exposure (0), low exposure (1–3), medium exposure (4–6) and high exposure (7–9). The method of contraceptive used had 3 categories: “no method”, “folk/traditional”, and “modern”.

***Distant***

Educational level was defined as “no education”, “primary”, and “secondary/higher”. Religion was grouped as “Catholics”, “other Christians”, “Islam” and “Traditionalist/other”. Respondents’ stated ethnicities were categorized into major ethnic groups of each region (North and South). Since the ethnic composition of the North and South were different, two variables for ethnicity were created and used only for the separate regional analyses. Type of residence was dichotomized as urban or rural. The NDHS measured wealth index with an asset score which was categorized in this study as poor, middle and rich. Water source was classified as improved or unimproved depending on whether there was a natural construction or a deliberate intervention to protect it from outside contamination.

Social autonomy was a composite variable that combined four measures of participation in decision making: woman’s involvement in decisions on her own health care, daily and large household purchases, and family/relative visits. Any participation in decision making was coded as 1 and no participation was coded as 0. The values for this composite variable ranged from 0 to 4, where 0 was no participation and a score of 4 meant participation in all four individual measures of decision making.

Similarly, attitude towards domestic violence was a composite variable that measured a participant’s attitude towards husband/partner beating wife in four circumstances: for going out without telling husband/partner, neglecting children, arguing with husband/partner and refusing sex. A response of “no” was coded as 0 and meant disapproval of domestic violence while “yes/don’t know” was coded as 1 and represented approval or lack of knowledge on whether domestic violence was justified. The sum of these individual measures (range: 0–4) were the response options for the composite variable where a score of 0 meant a negative attitude towards domestic violence while, a score of 4 was approval of violence for all the measures.

##### Community level

Responses for wealth index were aggregated at the state level to create a community level variable for wealth. For community wealth, individuals whose wealth index was poor, or middle were coded as 0. Those who were rich were coded as 1. The mean of these values was then assigned to individuals in the respective states. This value represented the proportion of rich women in each state in Nigeria.

##### Other variables

The 6 geo-political zones in Nigeria were the response options for the variable region. The 3 northern zones (North West, North Central and North East) were classified as North and the rest (South East, South West, and South South) were grouped as South. To control for secular trends, survey year was also included in this study with 2008 and 2013 as the response categories.

### Statistical analyses

Statistical analyses were performed using Stata SE13 [35]. The data were weighted to adjust for sampling design, non-responsiveness, stratification and clustering. Maternal mortality ratios for 2008 and 2013 were computed for each region (North and South). Survey adjusted simple and multivariable logistic regression analyses were used to investigate the association of the independent variables and maternal mortality in the North and South of Nigeria using pooled data for the North (2008 and 2013) and South (2008 and 2013). Statistical significance for all regressions performed was determined at *p* < 0.05.

## Results

### Descriptive analysis

There was a total of 51,492 women of childbearing age in this study (63.4% from the North and 36.6% from the South). Three percent of the sample consisted of maternal mortality cases of which, 751 and 810 maternal deaths were reported in 2008 and 2013 respectively.

### North

**Fig. 2:**
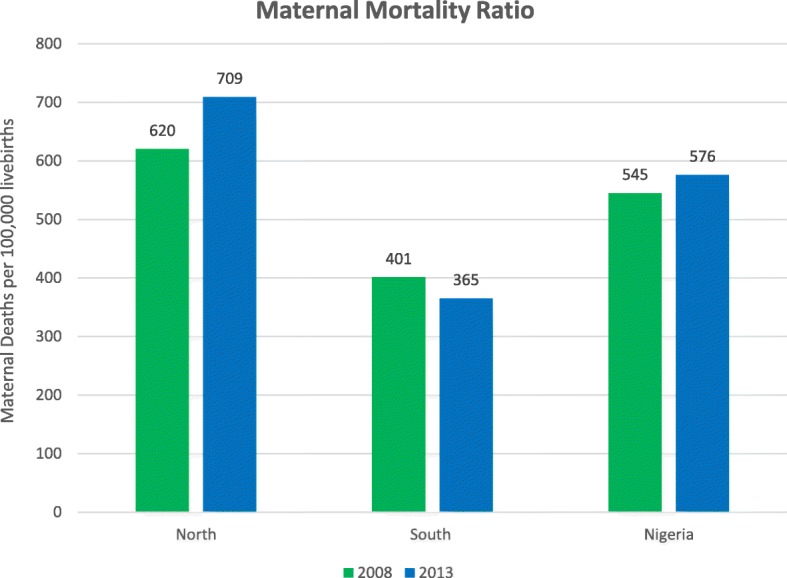
Maternal Mortality Ratio in the North, South, and Nigeria Overall - 2008 & 2013. Ratios for Nigeria were obtained from NDHS 2013 final report

The North contributed 32,629 women to the study sample. Most of the recorded maternal deaths (75.4%) were from this region which is also reflected in the MMR of the region (Fig. 2). The mean age of this sample was 30.1 years (Table 1). The majority of women (83.4%) had no or low media exposure, no education (66.1%) and were Muslim (75.4%).

**Table 1:** Characteristics of Women – North and South of Nigeria (2008 & 2013 pooled)

Variable	North (*n* = 32,629)	South (*n* = 18,863)	*p*-value
	n (%)	n (%)	
Survival status			
Alive	31,452 (96.4)	18,479 (97.9)	< 0.0001
Dead	1177 (3.6)	384 (2.0)	
	Mean (SD)	Mean (SD)	
Community level			
Community wealth (range: 0–1)	0.21 (0.15)	0.61 (0.18)	< 0.0001
Individual level			
Age (range: 15–49)	31.1 (8.77)	33.62 (8.4)	< 0.0001
Social autonomy (range: 0–4)	1.19 (1.52)	2.49 (1.38)	< 0.0001
Domestic violence (range: 0–4)	1.38 (1.65)	0.89 (1.35)	< 0.0001
Categorical variables	n (%)	n (%)	
Distance to facility			
Small problem	20,232 (62.3)	13,044 (69.4)	< 0.0001
Big problem	12,264 (37.7)	5754 (30.6)	
Media exposure			
No exposure	14,106 (43.3)	2754 (14.6)	
Low exposure	13,054 (40.1)	7353 (39.0)	< 0.0001
Medium exposure	4876 (14.9)	7619 (40.4)	
High exposure	559 (1.7)	1117 (5.9)	
Contraception type			
No method	30,232 (92.7)	13,540 (71.8)	
Folk/traditional	400 (1.2)	2044 (10.8)	< 0.0001
Modern	1997 (6.1)	2430 (18.6)	
Education			
No education	21,550 (66.1)	2189 (11.6)	
Primary	5530 (16.9)	5866 (31.1)	< 0.0001
Secondary/higher	5549 (17.1)	10,808 (57.3)	
Religion			
Catholics	1660 (5.1)	2946 (15.7)	
Other Christians	5844 (18.0)	12,897 (68.6)	< 0.0001
Islam	24,461 (75.4)	2606 (13.9)	
Traditionalist/Other	458 (1.4)	356 (1.9)	
Ethnicity North			
Fulani	3928 (12.1)	–	
Hausa	13,388 (41.2)	–	
Kanuri/Beriberi	1084 (3.3)	–	
Tiv	1091 (3.4)	–	
Yoruba	1160 (3.6)	–	
Others	11,848 (36.5)	–	< 0.0001
Ethnicity South			
Ekoi/Ibibio	–	1364 (7.3)	
Igbo	–	5522 (29.4)	
Ijaw/Izon	–	1863 (9.9)	
Yoruba	–	5695 (30.3)	
Others	–	4371 (23.2)	
Type of residence			
Urban	7450 (22.8)	9055 (48.0)	< 0.0001
Rural	25,179 (77.2)	9808 (52.0)	
Wealth index			
Poor	19,771 (60.6)	3096 (16.4)	
Middle	6164 (18.9)	4173 (22.1)	< 0.0001
Rich	6694 (20.5)	11,594 (61.5)	
Water quality			
Improved	15,580 (48.1)	11,501 (62.1)	< 0.0001
Unimproved	16,845 (51.9)	7032 (37.94)	
Year			
2008	15,499 (47.5)	8389 (44.5)	
2013	17,130 (52.5)	10,474 (55.5)	

### South

A total of 18,863 women were from the South with 24% of the reported maternal deaths (Fig. 2). They were an average age of 33.6 years. Most women (79.4%) had a low or medium level media exposure and a secondary or higher education (57.3%). The women from the South were mostly Christian (84.3%).

### Bivariate analyses (ref. Table 2)

#### North

In the North, community wealth, age and social autonomy were negatively associated with maternal mortality. Women who had low media exposure had increased odds of maternal mortality (reference: no exposure). Having a secondary or higher education reduced the odds of maternal death by 23% (reference: no education). Rich women had 20% lower odds of maternal death compared to those who were poor.

**Table 2:** Bivariate Analyses of Determinants of Maternal Mortality - North and South

Variable	North (*n* = 32,629) OR (95% CI)	South (*n* = 18,863) OR (95% CI)
Community level		
Community wealth	**0.17 (0.09–0.32)**	**0.36 (0.19–0.68)**
Individual		
Age	**0.91 (0.89–0.92)**	**0.92 (0.90–0.94)**
Social autonomy	**0.92 (0.87–0.98)**	0.98 (0.89–1.07)
Domestic violence	1.02 (0.98–1.06)	**1.10 (1.02–1.20)**
Distance to facility		
Small problem	1	1
Big problem	1.01 (0.87–1.16)	1.03 (0.78–1.34)
Media exposure		
No exposure	1	1
Low exposure	**1.18 (1.01–1.38)**	**1.52 (1.04–2.23)**
Medium exposure	0.93 (0.72–1.21)	1.40 (0.96–2.04)
High exposure	0.61 (0.32–1.14)	1.35 (0.75–2.43)
Contraception type		
No method	1	1
Folk/traditional	0.82 (0.41–1.62)	**1.48 (1.06–2.06)**
Modern	0.82 (0.58–1.15)	1.05 (0.76–1.46)
Education		
No education	1	1
Primary	0.83 (0.68–1.02)	1.49 (0.99–2.25)
Secondary/higher	**0.77 (0.63–0.95)**	1.47 (0.98–2.21)
Religion		
Catholics	1	1
Other Christians	0.94 (0.63–1.39)	0.90 (0.65–1.24)
Islam	1.35 (0.95–1.90)	0.70 (0.46–1.08)
Traditionalist/Other	1.32 (0.74–2.35)	0.88 (0.37–2.09)
Ethnicity - North		
Fulani	1	**–**
Hausa	0.83 (0.66–1.05)	**–**
Kanuri/Beriberi	0.94 (0.62–1.42)	**–**
Tiv	0.82 (0.56–1.20)	**–**
Yoruba	0.40 (0.23–0.71)	**–**
Others	0.70 (0.54–0.90)	**–**
Ethnicity - South		
Ekoi/Ibibio	**–**	1
Igbo	**–**	0.72 (0.46–1.11)
Ijaw/Izon	**–**	0.68 (0.38–1.22)
Yoruba	**–**	**0.41 (0.26–0.65)**
Others	**–**	**0.48 (0.30–0.76)**
Type of residence		
Urban	1	1
Rural	1.05 (0.87–1.28)	0.91 (0.72–1.15)
Wealth index		
Poor	1	1
Middle	0.99 (0.82–1.22)	1.32 (0.90–1.94)
Rich	**0.80 (0.65–0.98)**	1.13 (0.81–1.56)
Water quality		
Improved	1	1.00
Unimproved	1.07 (0.92–1.25)	1.08 (0.84–1.39)
Year		
2004	1	1
2011	0.99 (0.85–1.17)	0.94 (0.74–1.19)

Bold: statistical significance *p* < 0.05

Ethnicity: Two separate variables for ethnic composition were used for regional analyses

#### South

In the South, the odds of maternal death decreased with increasing age and community wealth. An increase in the score for attitude towards domestic violence increased the odds of maternal death by 10%. A low level of media exposure increased the odds of maternal mortality by 52% compared to women with no media exposure. Users of folk/traditional contraceptive methods had a 48% increase in their odds of maternal mortality (reference: no method). Among the southern ethnic groups, being Yoruba and others ethnic group was protective against maternal mortality (reference: Ekoi/Ibibio).

### Multivariable analyses (ref. Table 3)

In the North, the odds of maternal death reduced with increasing age in years by 9% and by 86% with an increase in community wealth. Women with low media exposure had 39% higher odds of maternal death compared to those with no media exposure. A secondary/higher education significantly reduced the odds of maternal mortality by 47% (reference: no education).

**Table 3:** Multivariable Analyses of Determinants of Maternal Mortality: North and South

Variable	North (n = 32,629) OR (95% CI)	South (n = 18,863) OR (95% CI)
Community level		
Community wealth	**0.14 (0.06–0.33)**	**0.38 (0.16–0.89)**
Individual		
Age	**0.91 (0.89–0.92)**	**0.90 (0.88–0.93)**
Social autonomy	1.01 (0.95–1.08)	1.06 (0.95–1.17)
Domestic violence	0.98 (0.94–1.03)	1.04 (0.92–1.16)
Distance to facility		
Small problem	1	1
Big problem	0.99 (0.84–1.17)	0.84 (0.59–1.19)
Media exposure		
No exposure	1	1
Low exposure	**1.39 (1.18–1.63)**	1.43 (0.89–2.29)
Medium exposure	1.14 (0.84–1.55)	1.51 (0.93–2.46)
High exposure	1.16 (0.55–2.46)	1.54 (0.70–3.36)
Contraception type		
No method	1	1
Folk/traditional	1.50 (0.73–3.08)	**1.75 (1.20–2.56)**
Modern	1.44 (0.98–2.10)	1.21 (0.84–1.77)
Education		
No education	1	1
Primary	0.82 (0.64–1.05)	1.02 (0.61–1.71)
Secondary/higher	**0.53 (0.39–0.74)**	0.63 (0.36–1.11)
Religion		
Catholics	1	1
Other Christians	1.07 (0.61–1.86)	0.96 (0.61–1.49)
Islam	1.13 (0.62–2.06)	0.99 (0.55–1.79)
Traditionalist/Other	1.19 (0.54–2.59)	1.16 (0.42–3.24)
Ethnicity - North		
Fulani	1	**–**
Hausa	0.82 (0.64–1.06)	**–**
Kanuri/Beriberi	1.04 (0.65–1.64)	**–**
Tiv	0.98 (0.53–1.79)	**–**
Yoruba	1.02 (0.52–1.99)	**–**
Others	0.99 (0.72–1.38)	**–**
Ethnicity - South		
Ekoi/Ibibio	**–**	1
Igbo	**–**	1.03 (0.61–1.76)
Ijaw/Izon	**–**	1.20 (0.63–2.32)
Yoruba	**–**	0.69 (0.40–1.19)
Others	**–**	0.69 (0.39–1.19)
Type of residence		
Urban	1	1
Rural	0.90 (0.69–1.16)	**0.68 (0.51–0.92)**
Wealth index		
Poor	1	1
Middle	1.11 (0.88–1.39)	**1.75 (1.04–2.94)**
Rich	1.14 (0.84–1.54)	1.56 (0.92–2.64)
Water quality		
Improved	1	1.00
Unimproved	1.01 (0.84–1.20)	1.14 (0.84–1.55)
Year		
2008	1	1
2013	0.97 (0.82–1.15)	0.90 (0.67–1.19)

Bold: statistical significance *p* < 0.05

Ethnicity: Two separate variables for ethnic composition were used for regional analyses

Like the North, age and community wealth were also significant in the South, where the odds of maternal mortality decreased by 10% with increasing age in years and by 62% with increasing community wealth. Users of folk/traditional methods of contraception had 75% high odds of maternal death compared to nonusers. In the South, being a rural resident was protective against maternal mortality with a 32% reduction in the odds of maternal death. The middle class, compared to the poor, had higher odds of maternal death.

## Discussion

The North and South of Nigeria differed in their levels of maternal mortality. Maternal deaths increased in the North and slightly decreased in the South between the years 2008 and 2013. While the North and South had similarities in terms of determinants of maternal mortality (age and community wealth), in the North, there were significant associations between media exposure, level of education, and maternal mortality. In the South, contraceptive method used, type of residence, and wealth index were significantly associated with maternal mortality.

### Maternal mortality levels in the North and South

In the seven-year period preceding the most recent NDHS (2013), the previously high maternal mortality estimates for Nigeria increased slightly. Our study explored the levels of maternal mortality for the North and South regions separately. The North had more maternal deaths than the south. Over time, the North saw an increase in the levels of maternal mortality while the South had a minor decrease in its levels.

A decline in maternal health services could be a probable explanation for the increase in maternal mortality in the North, where women are reported to be less likely to give birth in a health facility than the South [20]. The insufficient and poor quality of health services in the region, health worker shortage, and substandard emergency obstetric care services [18] owing to the elimination of grants for health institutions, [4] may have increased the cost of health care (60% of women in the North are poor) and increased the proportion of deliveries outside the health facility. Previous findings in the northern state of Kebbi show that after community education on emergency health services, their use did not increase even though awareness about them improved [36].

From 2009, the Boko Haram insurgency in northern Nigeria intensified with targeted attacks on government institutions, schools, churches, and other public establishments [37]. Some residents became internally displaced or refugees in neighboring countries. Diarrhea and malnutrition were commonly recorded among displaced persons living in poor conditions with restricted access to health and other basic services. Maternal deaths in the camps were due primarily to excessive bleeding [38]. Reports show that non-indigenous health workers fled from conflict zones to safer states while insurgents destroyed and removed medical supplies from health facilities [39].

This conflict possibly influenced the level of maternal mortality in the North in 2013 compared to that of 2008 due to its devastating impact on the health system. For instance, Borno state capital received several internally displaced persons while one of its prominent health facilities recorded 76 maternal deaths within 6 months in 2009 [40]. This may indicate a strain on the health system which in turn affects the health of people already adversely affected by the conflict.

### Determinants of maternal mortality

#### North

The odds of maternal mortality decreased with increasing age, placing the greatest odds of maternal death on young women. Evidence from other studies show varying associations between age and maternal mortality. The risk of maternal death was found to be greatest for teenage mothers and women close to the end of their childbearing years [4]. Other studies found older women to have the greatest risk of maternal death [41, 42]. In the North, early marriages expose young girls to early childbearing and pregnancies that are prone to complications and may lead to maternal death. This is further aggravated by cultural practices in the region that prevent pregnant women from seeking care or showing signs of distress from the pregnancy [24].

Community wealth influences availability and access to maternal health services. Wealthy communities have better access to resources and services compared to poor ones. Community wealth was associated with maternal mortality in the North meaning that states with high proportions of wealthy women had reduced odds of maternal death compared to those states that consisted mostly of poor or middle-class women. This finding is consistent with other studies. Higher community income was linked with fewer maternal deaths in Madagascar [43]. In Rwanda, high community wealth was associated with better access to maternal health services [44]. Maternal mortality in the North may be influenced by low community wealth given the low proportion (21%) of wealthy women in northern states.

Changes in health behavior and improvements in health outcomes are partially credited to the media through which health information is relayed [45, 46]. In Nigeria, any media exposure, compared to no exposure was positively associated with antenatal care utilization in adolescent women [47]. However, in this study, women who had low media exposure had higher odds of experiencing maternal mortality compared to those with none. This reveals variations in the influence of the different levels of media exposure that may have been masked in the results seen by Rai, Singh, & Singh (2012) where the different levels of media exposure were aggregated. Low access to information may be contributing to the low level of health service utilization and a high proportion of women who do not use any antenatal care services in the North [48] which influence maternal mortality.

Secondary or higher education was protective against maternal mortality in the North compared to having no education. This is consistent with other studies [4, 41, 49]. In one study, nonusers of maternal health services were more likely to be less educated [48]. The risk of maternal death may be higher in women with less education because of factors such as early marriage and other cultural practices that restrict their access and participation in the labor force. Wall (1998) revealed strict conditions in the North that undermine women’s autonomy and influence their risk of maternal death. This is most evident in the large proportion (66%) of women with no education in the region. Meanwhile, highly educated women tend to use and demand maternal health services [50].

#### South

Like the North, age and community wealth were significantly associated with maternal mortality in the South. The odds of maternal death decreased with increasing age and increasing community wealth. In contrast, some factors were only significantly associated with maternal mortality in the South. These were contraceptive method used, residence type and wealth.

The method of contraception used in the South was associated with maternal mortality. The likelihood of dying was significantly higher in women who used folkloric or traditional methods compared to nonusers. Nonusers may want pregnancies, while users intend to prevent them. Using traditional contraceptives may not effectively prevent unwanted pregnancies as much as modern methods would. Unwanted pregnancies expose women to unsafe abortions that are linked to maternal deaths. In the South, hemorrhage and illegal abortions were the leading causes of maternal death [4]. It has been suggested that women in the South rely on abortions to maintain smaller family sizes [51]. Women may choose traditional/folkloric over modern contraceptives for reasons such as the fear of side effects, which is a known barrier to contraceptive use [52, 53].

A rural residence was protective against maternal mortality for women in the South. This was unlike other studies that show an increased risk of maternal mortality for women living in rural areas compared to those in urban settings [4]. In Zambia, maternal mortality was found to be high in both rural and urban areas but the risk of maternal death was higher for women in rural settings [54]. Because access to care is usually better in urban than rural areas, this finding may imply that the quality of health services such as emergency obstetrics services in the urban areas of the South is poor. Practices such as unsafe abortions among women and a high prevalence of infectious diseases like HIV that are linked with maternal mortality may explain high mortality levels in urban areas [1]. These may explain the problem of maternal mortality in the urban settings of the South compared to rural ones.

The middle class, compared to poor women, were more likely to experience maternal mortality in the South region. The risk of maternal death may be high among the middle class because they are more likely to seek care at health facilities compared to the poor. Their choice of facilities may be less expensive but lacking in terms of service quality. A review study of place of delivery in sub-Saharan Africa revealed that women who gave birth at health facilities had a higher risk of maternal death than women who gave birth at home [55]. This may imply that women who do seek care receive substandard services in these health facilities. There may also be other health risks in women of the middle class such as abortions. In Bangladesh, induced abortions were more common among educated women [41] although the risk of mortality was low for them compared to uneducated women, this may not be the case in the South of Nigeria, where abortion is restricted, and most of its services are illicit and unsafe.

### Policy recommendations

Nigeria still has a high MMR and needs to reevaluate the already established programs to reduce maternal mortality in the country. This is especially needed in the North where interventions (material, staffing and educational activities) have been applied to the health system [11, 18, 36] yet, the MMR for the region remains high and has slightly increased over time. Reducing maternal mortality in the North calls for more focused interventions. The North has historically had varied barriers (cultural, financial, physical and boycotts) that prevent the utilization of services that could improve maternal and other health outcomes in the region [56]. Hence, new and current strategies need to be sensitive to the environmental of the women in the North.

Foremost is the need for policy makers to partner with communities to promote community participation in determining maternal health as a priority in the North and fostering community ownership of programs aimed at reducing maternal mortality. These programs need to be innovative in their efforts to ensure that maternal health services are available, affordable, acceptable and utilized within and outside health facilities. As many women in the North continue to give birth unattended outside health facilities [57], the government of Nigeria, together with community leaders in the North, need to tackle this by identifying members of the community that can be trained as local midwives and birth attendants. These workers can offer basic services to pregnant women in their homes and provide information on safe motherhood practices and where to get help.

Women in northern Nigeria are increasingly vulnerable to maternal mortality due in part to the adverse effects of Boko Haram’s activities. For communities to tackle maternal mortality, stability and security need to be restored in the North for both indigenous populations and stakeholders to function at full capacity. The government must intensify its efforts towards providing maternal health services in a conflict zone using alternate strategies that do not impinge on the already fragile healthcare institution. Some counter-insurgency tactics have been restrictive in terms of health services provision and access [39].

Child marriage, which is commonplace in the North, promotes early childbearing, exposes young women to birth complications due to the immature nature of their bodies and prevents the education of girls. This is so even with Nigeria being a signatory member for the Universal Declaration of Human Rights and the African Charter on the Rights and Welfare of the Child. With over 80% of women with little or no media exposure in the North, this means that information about the benefits of education and health information do not reach most who stand to benefit from them.

It is thus necessary to gain community support to increase the legal age of marriage for girls in the North. This can be achieved through innovative means of media communication and new strategies for education which will encourage school attendance in the North. Even though Nigeria ratified the convention on the rights of the child, the problem has persisted in the region. Hence, the government needs to call all stakeholders to recommit to the fight against child marriages and clearly delineate their roles. Meanwhile, they should also coordinate and properly structure interventions to prevent child marriages.

The South saw a slight decline in its MMR but there remains a need for the government to ensure that there are appropriate family planning services and education available in the South. Further research is needed to understand why women in urban areas compared to those in rural areas experienced more maternal deaths. Overall, policy makers should seek to increase maternal age and alleviate poverty by improving the economic conditions of women in the country.

### Limitations

There was limited information obtained from the survey respondents about deceased siblings in the 2008 and 2013 NDHS. This study thereby ascribed respondent characteristics to the deceased siblings to allow for comparisons on more determinants of maternal mortality. This was on the assumption that deceased siblings share similar characteristics as the respondents. It is possible that some deceased siblings had different characteristics compared to those ascribed to them which may lead to the misclassification of their own attributes.

Nonetheless, sociodemographic characteristics of living women are often similar to those of their deceased siblings. Evidence from the literature supports this approach of using respondent characteristics as proxy for deceased sibling in the assessment of maternal mortality [26, 33, 34] particularly in settings where cases of maternal mortality cannot be directly evaluated because of limited data on the deceased. The causes of maternal death cannot be ascertained using this method or DHS data.

Using DHS data to examine maternal mortality has its limitations. Though estimates of maternal mortality levels can be derived with the Sisterhood method, these estimates are not current in terms of representing the survey year in which the data were collected. They represent the level of maternal mortality over a period usually 5 to 7 years prior to the survey. It does not allow for the estimation of annual rates that could show variations in maternal mortality levels across time.

More so, maternal mortality levels obtained from DHS data underestimate the magnitude of the problem. The information obtained is based on respondents’ knowledge of sibling’s pregnancy status. Cases of maternal mortality may be missed where death occurred in early pregnancy or deliberately not reported by respondents because of sensitive issues like abortion complications.

The modified framework retained some determinants of maternal mortality from the McCarthy and Maine framework. Others were omitted because they were unavailable in the data. Community education and parity were excluded from this study because they were highly correlated with community wealth and age respectively, and parity was not reported in NDHS 2008. Since their effects were adequately captured with community wealth and age, the latter were used as their proxies. Hence, the findings of this study should be interpreted in light of these limitations.

## Conclusion

The findings of this study show that maternal mortality remains a problem in Nigeria and reveal its levels and determinants in the North and South. In the North, maternal mortality has increased, and this is associated with media exposure and education. In the South, it has slightly decreased and is associated with contraceptive method used, type of residence and wealth. These differences between the North and South of Nigeria have implications for maternal health programs to prevent maternal deaths. Though education and economic advancement for women and young girls are central to the improvement of maternal health in the country, the importance of region specific factors on the occurrence of maternal mortality require interventions that explicitly target their influence in the respective regions.

## Data Availability

The 2008 and 2013 Nigeria Demographic and Health Survey datasets used for this study are publicly available through the Measure DHS program https://dhsprogram.com/data/available-datasets.cfm and the formats used in this study are available upon request to the corresponding author.

## Abbreviations

CI: Confidence Interval
MDG: Millennium Development Goal
MMR: Maternal Mortality Ratio
NDHS: Nigeria Demographic and Health Survey
OR: Odds Ratio
SDG: Sustainable Development Goal
USAID: United States Agency for International Development

## References

[1] Ronsmans C, Graham WJ (2006). Maternal survival 1: maternal mortality: who, when, where, and why. Lancet.

[2] WHO. WHO maternal mortality: WHO. World Health Organization; 2016. Available from: https://www.who.int/en/news-room/fact-sheets/detail/maternal-mortality. Cited 28 June 2017

[3] Khan KS, Wojdyla D, Say L, Gülmezoglu AM, Van Look PF (2006). WHO analysis of causes of maternal death: a systematic review. Lancet.

[4] Adamu YM, Salihu HM, Sathiakumar N, Alexander GR (2003). Maternal mortality in northern Nigeria : a population-based study. Eur J Obstet Gynecol Reprod Biol.

[5] WHO (2001). Maternal mortality in 1995: estimates developed by WHO, UNICEF, UNFPA.

[6] Alkema L, Chou D, Hogan D, Zhang S, Moller A-B, Gemmill A (2016). Global, regional, and national levels and trends in maternal mortality between 1990 and 2015, with scenario-based projections to 2030: a systematic analysis by the UN maternal mortality estimation inter-agency group. Lancet (London, England). NIH Public Access.

[7] WHO (2014). Trends in maternal mortality: 1990 to 2013. Estimates by WHO, UNICEF, UNFPA.

[8] Hogan MC, Foreman KJ, Naghavi M, Ahn SY, Wang M, Makela SM (2010). Maternal mortality for 181 countries, 1980-2008: a systematic analysis of progress towards millennium development goal 5. Lancet (London, England). Elsevier.

[9] WHO (2010). Trends in maternal mortality: 1990 to 2008.

[10] National Population Commission (NPC) [Nigeria] and ICF International. Nigeria Demographic and Health Survey 2013. Abuja and Rockville: NPC and ICF International; 2014. https://dhsprogram.com/pubs/pdf/FR293/FR293.pdf.

[11] Galadanci H, Idris S, Sadauki H, Yakasai I (2010). Programs and policies for reducing maternal mortality in Kano state Nigeria: a review. Afr J Reprod Health.

[12] Doctor HV, Olatunji A, Findley SE, Afenyadu GY, Abdulwahab A, Jumare A (2012). Maternal mortality in northern Nigeria: findings of a health and demographic surveillance system in Zamfara state, Nigeria. Trop Dr.

[13] Fawole AO, Shah A, Fabanwo AO, Adegbola O, Adewunmi AA, Eniayewun AB (2012). Predictors of maternal mortality in institutional deliveries in Nigeria. Afr Health Sci.

[14] Ujah IAO, Aisien OA, Mutihir JT, Vanderjagt DJ, Glew RH, Uguru VE (2005). Factors contributing to maternal mortality in North-Central Nigeria: A seventeen-year review. Afr J Reprod Health.

[15] Lanre-Abass BA (2008). Poverty and maternal mortality in Nigeria: towards a more viable ethics of modern medical practice. Int J Equity Health.

[16] Akinlo A, Idemudia ES, Ogunjuyigbe PO, Solanke BL (2016). Women’s empowerment status and exposure to maternal mortality risks in Nigeria. Gend Behav.

[17] Adebowale AS, Yusuf BO, Fagbamigbe AF (2012). Survival probability and predictors for woman experience childhood death in Nigeria: analysis of north-south differentials. BMC Public Health.

[18] Doctor HV, Findley SE, Ager A, Cometto G, Afenyadu GY, Adamu F (2012). Using community-based research to shape the design and delivery of maternal health services in northern Nigeria. Source Reprod Heal Matters.

[19] McDermott R, Cowden J. Polygyny and violence against women. Emory Law J. 2015;64(6):1767-814. http://law.emory.edu/elj/content/volume-64/issue-6/articles-and-essays/polygyny-violence-against-women.html.

[20] Ononokpono DN, Odimegwu CO (2014). Determinants of maternal health care utilization in Nigeria: a multilevel approach. Pan Afr Med J.

[21] Adebayo SB, Gayawan E, Ujuju C, Ankomah A (2013). Modelling geographical variations and determinants of use of modern family planning methods among women of reproductive age in Nigeria. J Biosoc Sci.

[22] Babalola S, Oyenubi O (2018). Factors explaining the north-south differentials in contraceptive use in Nigeria: a nonlinear decomposition analysis. Demogr Res.

[23] Eboreime E, Abimbola S, Bozzani F (2015). Access to routine immunization: a comparative analysis of supply-side disparities between northern and southern Nigeria. PLoS One.

[24] Wall LL (1998). Dead mothers and injured wives: the social context of maternal morbidity and mortality among the Hausa of northern Nigeria. Stud Fam Plan.

[25] McCarthy J, Maine D (1992). A framework for analyzing the determinants of maternal mortality. Stud Fam Plan.

[26] Ariyo O, Ozodiegwu ID, Doctor HV (2017). The influence of the social and cultural environment on maternal mortality in Nigeria: evidence from the 2013 demographic and health survey. PLoS One.

[27] Wall LL (2012). A framework for analyzing the determinants of obstetric fistula formation. Stud Fam Plan.

[28] Kusuma D, Cohen J, McConnell M, Berman P (2016). Can cash transfers improve determinants of maternal mortality? Evidence from the household and community programs in Indonesia. Soc Sci Med.

[29] Graham W, Brass W, Snow RW (1989). Estimating maternal mortality: the sisterhood method. Stud Fam Plan.

[30] Merdad L, Hill K, Graham W (2013). Improving the measurement of maternal mortality: the sisterhood method revisited. PLoS One.

[31] Betrán AP, Wojdyla D, Posner SF, Gülmezoglu AM (2005). National estimates for maternal mortality: an analysis based on the WHO systematic review of maternal mortality and morbidity. BMC Public Health.

[32] Hill K, Thomas K, AbouZahr C, Walker N, Say L, Inoue M (2007). Estimates of maternal mortality worldwide between 1990 and 2005: an assessment of available data. Lancet Elsevier.

[33] El Ayadi AM, Hill K, Langer A, Subramanian SV, McCormick M (2015). Comparability of sociodemographic and pregnancy characteristics of pregnancy-related deaths identified via the sisterhood method versus the household/verbal autopsy method. Int J Gynecol Obstet.

[34] Graham WJ, Fitzmaurice AE, Bell JS, Cairns JA (2004). The familial technique for linking maternal death with poverty. Lancet.

[35] Stata Corp (2013). Stata Statistical Software: Release 13. 2013.

[36] Bello Gummi F, Hassan M, Shehu D, Audu L (1997). Community education to encourage use of emergency obstetric services, Kebbi state, Nigeria. Int J Gynecol Obstet.

[37] Agbiboa DE, Maiangwa B (2013). Boko haram, religious violence, and the crisis of National Identity in Nigeria: towards a non-killing approach. J Dev Soc.

[38] Omole O, Welye H, Abimbola S (2015). Boko Haram insurgency: Implications for public health. Lancet.

[39] Ager AK, Lembani M, Mohammed A, Mohammed Ashir G, Abdulwahab A, De Pinho H (2015). Health service resilience in Yobe state, Nigeria in the context of the Boko haram insurgency: a systems dynamics analysis using group model building. Confl Heal.

[40] Adamu PI, Adamu MO, Okagbue HI (2018). Data in support of high rate of pregnancy related deaths in Maiduguri, Borno state, Northeast Nigeria. Data Br.

[41] Chowdhury ME, Botlero R, Koblinsky M, Saha SK, Dieltiens G, Ronsmans C (2007). Determinants of reduction in maternal mortality in Matlab, Bangladesh: a 30-year cohort study. Lancet.

[42] Kassebaum NJ, Bertozzi-Villa A, Coggeshall MS, Shackelford KA, Steiner C, Heuton KR (2014). Global, regional, and national levels and causes of maternal mortality during 1990–2013: a systematic analysis for the global burden of disease study 2013. Lancet.

[43] Hernandez JC, Moser CM (2013). Community level risk factors for maternal mortality in Madagascar. Afr J Reprod Health.

[44] Stephenson R, Elfstrom KM (2012). Community influences on antenatal and delivery care in Bangladesh, Egypt, and Rwanda. Glob Heal Matters Public Heal Reports.

[45] Choe SA, Kim J, Kim S, Park Y, Kullaya SM, Kim CY (2016). Do antenatal care visits always contribute to facility-based delivery in Tanzania? A study of repeated cross-sectional data. Health Policy Plan.

[46] Wakefield MA, Loken B, Hornik RC (2010). Use of mass media campaigns to change health behaviour. Lancet.

[47] Rai RK, Singh PK, Singh L (2012). Utilization of maternal health care services among married adolescent women: insights from the Nigeria demographic and health survey, 2008. Womens Health Issues.

[48] Fagbamigbe AF, Idemudia ES (2015). Barriers to antenatal care use in Nigeria : evidences from non-users and implications for maternal health programming. BMC Pregnancy Childbirth.

[49] Karlsen S, Say L, Souza J-P, Hogue CJ, Calles DL, Gülmezoglu AM (2011). The relationship between maternal education and mortality among women giving birth in health care institutions: analysis of the cross sectional WHO global survey on maternal and perinatal health. BMC Public Health.

[50] Aremu O, Lawoko S, Dalal K (2011). Neighborhood socioeconomic disadvantage, individual wealth status and patterns of delivery care utilization in Nigeria: a multilevel discrete choice analysis. Int J Women's Health.

[51] Bankole A, Adewole IF, Hussain R, Awolude O, Singh S, Akinyemi JO (2015). The incidence of abortion in Nigeria. Int Perspect Sex Reprod Health.

[52] Durowade KA, Omokanye LO, Elegbede OE, Adetokunbo S, Olomofe CO, Ajiboye AD (2017). Barriers to contraceptive uptake among women of reproductive age in a semi-Urban Community of Ekiti state, Southwest Nigeria. Ethiop J Health Sci.

[53] Oluwole E, Kuyinu Y, Goodman O, Odugbemi B, Akinyinka M (2016). Factors influencing the uptake of modern family planning methods among women of reproductive age in a rural Community in Lagos State. Int J Trop Dis Heal.

[54] Banda R, Fylkesnes K, Sandøy IF (2015). Rural-urban differentials in pregnancy-related mortality in Zambia: estimates using data collected in a census. Popul Health Metrics.

[55] Chinkhumba J, De Allegri M, Muula AS, Robberstad B (2014). Maternal and perinatal mortality by place of delivery in sub-Saharan Africa: a meta-analysis of population-based cohort studies. BMC Public Health.

[56] McArthur-Lloyd A, McKenzie A, Findley SE, Green C, Adamu F (2016). Community engagement, routine immunization, and the polio legacy in northern Nigeria. Glob Heal Commun.

[57] Fapohunda BM, Orobaton NG (2013). When women deliver with no one present in Nigeria: who, what, where and so what?. PLoS One.

